# Assessment of esophageal motility in patients with eosinophilic esophagitis: A scoping review

**DOI:** 10.1097/MD.0000000000049950

**Published:** 2026-07-24

**Authors:** Ali Karim, Rabia Amir, Muhammad Bari Hassan, Mubeena Javed, Mahmood ul Hassan Akhundzada, Danish Kumar Goswami, Alfa Almani, Sajid Ali, Hem Raj, Aamer R. Mohammad, Abida Perveen

**Affiliations:** a Department of Medicine, Ibn e Seena Hospital, Kabul, Afghanistan.

**Keywords:** dysphagia, eosinophilic esophagitis, esophageal distensibility, esophageal motility, FLIP panometry, high-resolution manometry

## Abstract

Eosinophilic esophagitis (EoE) is a chronic, immune-mediated disorder characterized by eosinophilic infiltration of the esophageal mucosa, leading to symptoms such as dysphagia and food impaction. While histologic and endoscopic features are well established, the impact of EoE on esophageal motility has gained increasing attention. This narrative review evaluates current evidence on esophageal motility abnormalities in EoE, focusing on diagnostic modalities including high-resolution manometry (HRM), functional lumen imaging probe (FLIP), and impedance measurements. Chronic inflammation and subepithelial fibrosis in EoE alter esophageal biomechanics, resulting in diverse motility abnormalities such as ineffective esophageal motility, fragmented peristalsis, panesophageal pressurization, and occasional achalasia-like patterns. HRM has emerged as a key tool for detecting these abnormalities, although interpretation remains challenging due to heterogeneity and lack of disease-specific patterns. Advanced techniques such as FLIP and impedance measurements provide additional insights by assessing esophageal distensibility and bolus transit, demonstrating reduced compliance and impaired bolus clearance that correlate with symptom severity and risk of food impaction. Esophageal motility assessment is essential for the comprehensive evaluation of EoE, particularly in patients with persistent or refractory symptoms. Integration of functional, histologic, and clinical findings is crucial for individualized management. Further research is needed to standardize diagnostic criteria and optimize therapeutic strategies based on motility assessment.

## 1. Introduction

Eosinophilic esophagitis (EoE) is a chronic, immune-mediated esophageal disorder characterized by eosinophilic infiltration of the esophageal epithelium, leading to symptoms of esophageal dysfunction such as dysphagia and food impaction.^[1]^ While the histopathological hallmark of EoE is well established, increasing attention has been directed toward the assessment of esophageal motility in these patients.^[2]^ Esophageal motility abnormalities, which may result from chronic inflammation, remodeling, and fibrosis, can significantly impact symptom presentation and disease progression.^[3]^ High-resolution manometry (HRM) and other motility studies have emerged as valuable tools to elucidate these functional alterations.^[4]^ In this narrative review, we explore the role of esophageal motility assessment in patients with EoE, discussing current evidence, diagnostic techniques, clinical implications, and the potential for integrating motility findings into comprehensive disease management.^[5]^

## 2. Discussion

### 2.1. Assessment of esophageal motility in patients with eosinophilic esophagitis

#### 2.1.1. Pathophysiology of EoE and its impact on esophageal motility

Eosinophilic esophagitis (EoE) is a chronic, antigen-driven inflammatory disease marked by eosinophilic infiltration of the esophageal epithelium.^[6]^ Over time, persistent inflammation leads to tissue remodeling, subepithelial fibrosis, and changes in smooth muscle function, all of which can disrupt normal esophageal motility.^[3]^ These pathophysiological changes are believed to contribute to the hallmark symptoms of EoE, including dysphagia and food impaction.^[7]^ The inflammatory cascade involves not only eosinophils but also mast cells, T-helper 2 (Th2) lymphocytes, and cytokines such as IL-5 and IL-13, all of which contribute to tissue damage and fibrosis.^[8]^

Remodeling results in rigidity of the esophageal wall and a reduction in compliance, which alters esophageal biomechanics.^[9]^ These changes are central to motility abnormalities, although the exact mechanisms are not fully understood.^[10]^ The interaction between inflammation, neuromuscular control, and structural integrity is complex, and motility dysfunction may occur even in the absence of visible structural strictures.^[11]^ This highlights the importance of functional assessment tools like manometry in understanding EoE beyond its histological characteristics.^[12]^

Importantly, although EoE predominantly affects the mucosal and submucosal layers, emerging evidence suggests that chronic inflammation and fibrosis may exert secondary effects on deeper layers, including smooth muscle function and neural regulation. However, a key unresolved question is whether the observed motility abnormalities represent true primary neuromuscular dysfunction or are secondary to mechanical consequences of esophageal wall stiffening and reduced compliance. This distinction is critical when interpreting motility findings in EoE.

### 2.2. Esophageal manometry in EoE: historical context and advancements

Historically, esophageal manometry using conventional techniques yielded limited insight into EoE-related motility changes.^[13]^ With the advent of high-resolution manometry (HRM), however, a more refined and objective characterization of esophageal pressure patterns became possible.^[14]^ HRM allows visualization of esophageal pressure in a topographic format, offering detailed assessment of esophageal peristalsis and esophagogastric junction (EGJ) function.^[15]^

Initial studies using HRM revealed that motility disorders in EoE are not only common but also varied in presentation.^[16]^ Unlike primary motility disorders such as achalasia or diffuse esophageal spasm, motility abnormalities in EoE are often subtle and heterogeneous.^[17]^ These can range from ineffective esophageal motility (IEM) and weak peristalsis to panesophageal pressurization and even patterns mimicking achalasia. Despite this variability, HRM has become an essential investigative tool, particularly when patients report persistent dysphagia despite histologic remission or dilation of strictures.^[18]^

However, it is important to recognize that HRM findings in EoE must be interpreted with caution. Given that EoE is primarily a mucosal/submucosal disease, some manometric abnormalities—particularly those resembling achalasia—may represent secondary or “pseudoachalasia-like” patterns resulting from severe subepithelial fibrosis and reduced esophageal compliance rather than true primary motility disorders. This distinction underscores the need for careful clinicopathological correlation.

### 2.3. Prevalence and types of motility disorders in EoE

Multiple studies have attempted to quantify the prevalence of esophageal motility disorders in EoE. Reported abnormalities vary widely, occurring in 25% to 70% of patients, depending on study design and diagnostic criteria.^[19]^ This variability underscores the complexity of motility dysfunction in EoE and the influence of disease stage and prior treatment on motility findings^[20]^ as mentioned in table 1.

**Table 1:** Common esophageal motility abnormalities in EoE (with Chicago classification).

Motility abnormality	HRM diagnosis (Chicago Classification v4.0)	Description	Clinical significance
Ineffective esophageal motility (IEM)	Ineffective Esophageal Motility (IEM)	Low-amplitude or failed peristalsis in > 50% of swallows	Most common finding; associated with dysphagia and impaired bolus transit
Fragmented peristalsis	Minor disorder of peristalsis (fragmented contractions)	Disrupted or noncontinuous peristaltic waves	Reflects impaired neuromuscular coordination
Panesophageal pressurization	Seen in Type II achalasia pattern or compartmentalized pressurization (not specific alone)	Uniform pressurization of the entire esophagus during swallowing	Suggests reduced esophageal compliance, often due to fibrosis
Esophagogastric junction outflow obstruction (EGJOO)	EGJ Outflow Obstruction (EGJOO)	Incomplete LES relaxation with preserved peristalsis	May mimic achalasia; requires further evaluation with imaging/biopsy
Achalasia-like pattern	Does not always meet full Chicago criteria for achalasia	Aperistalsis with impaired LES relaxation	Rare in EoE; must be differentiated from true achalasia

The most frequently reported motility abnormality is ineffective esophageal motility (IEM), characterized by failed or weak peristaltic contractions. Other observed patterns include^[21]^:

- Fragmented peristalsis: Partial, discoordinated contractions that fail to propagate effectively^[22]^
- Panesophageal pressurization: Uniform pressurization along the esophagus during swallowing, often seen in the context of subepithelial fibrosis or reduced esophageal compliance^[23]^
- Esophagogastric junction outflow obstruction (EGJOO): Impaired relaxation of the lower esophageal sphincter (LES) without classic features of achalasia^[24]^
- Achalasia-like patterns: Although rare, EoE can present with complete aperistalsis and LES dysfunction mimicking primary achalasia^[24]^

It remains unclear whether these motility patterns are primary manifestations of EoE or secondary changes due to chronic inflammation and fibrosis. In particular, patterns such as panesophageal pressurization and achalasia-like findings may reflect impaired esophageal distensibility and luminal stiffness rather than intrinsic neuromuscular dysfunction. Nevertheless, recognition of these abnormalities is critical in guiding appropriate therapeutic decisions.^[25]^

### 2.4. Diagnostic challenges and the role of HRM

Interpreting HRM findings in EoE can be challenging due to the nonspecific and variable nature of the abnormalities.^[26]^ Unlike well-defined motility disorders categorized by the Chicago Classification, motility in EoE often presents with a mix of features that do not meet strict diagnostic criteria.^[27]^ Therefore, manometric findings must be interpreted in conjunction with clinical presentation, endoscopic findings, and histopathological results.^[27]^

One of the key challenges is distinguishing motility abnormalities due to EoE from those caused by coexisting or misdiagnosed motility disorders. For example, EoE may mimic or coexist with achalasia, making accurate diagnosis difficult without histological confirmation. In such cases, performing esophageal biopsies during endoscopy is essential to avoid misclassification.^[17]^

Furthermore, the possibility of pseudoachalasia-like physiology due to subepithelial fibrosis should be considered, particularly in patients with reduced esophageal compliance and preserved neural integrity. Adjunctive tools such as functional lumen imaging probe (FLIP) can be particularly useful in differentiating mechanical restriction from true motility disorders.

Additionally, the impact of treatment on motility findings must be considered. Studies have shown that corticosteroid therapy or dietary management can lead to partial or complete normalization of esophageal motility. Hence, timing of HRM in relation to therapy can influence results, and repeat testing may be necessary in some cases.^[28]^

### 2.5. Advanced motility assessment: FLIP and impedance measurements

In recent years, tools beyond traditional manometry have emerged to provide complementary information about esophageal function in EoE.^[29]^ One such tool is FLIP panometry, which assesses esophageal distensibility and compliance.^[30]^ FLIP uses impedance planimetry during volume-controlled distension of a balloon catheter to measure cross-sectional area and intraluminal pressure along the esophagus.^[31]^

FLIP studies in EoE have consistently shown decreased esophageal distensibility, which correlates with disease severity and subepithelial fibrosis.^[32]^ Reduced distensibility may be present even in the absence of strictures on endoscopy or barium studies, serving as a sensitive marker of remodeling. Importantly, FLIP findings have prognostic significance, as lower distensibility is associated with an increased risk of food impaction and resistance to dilation.^[33]^

These findings support the concept that impaired esophageal compliance plays a central role in symptom generation and may explain HRM patterns that resemble motility disorders. Thus, FLIP provides critical complementary information by distinguishing between mechanical stiffness and true neuromuscular dysfunction.

Impedance planimetry also offers the advantage of assessing esophageal bolus transit, which may be impaired in EoE despite preserved peristalsis.^[34]^ Combined with HRM, impedance manometry allows for comprehensive evaluation of esophageal clearance and bolus transport, providing further insight into symptom generation as shown in table 2.^[35]^

**Table 2:** High-resolution manometry vs FLIP in the evaluation of EOE.

Feature	High resolution manometry (HRM)	Functional lumen imaging probe (FLIP)
Purpose	Evaluate peristalsis and LES function	Measure esophageal distensibility and wall stiffness
Visualization	Topographic pressure plots	Cross -sectional area and pressure during ballon distension
Detects motility abnormality	Yes	Inderictly through abnormal distensibility pattern
Assess esophageal compliance	No	Yes
Clinical use in EoE	Identification dysmotility contribution to symptoms	Detects fibrosis related stiffness in histologic remission
Complementary	Diagnostic of motor pattern	Functional assessment of remodeling

### 2.6. Clinical implications and therapeutic relevance

Understanding esophageal motility in EoE has direct implications for patient management. Persistent dysphagia in EoE patients who are histologically in remission may be explained by underlying motility dysfunction or reduced distensibility.^[7]^ In such cases, therapeutic decisions must be tailored accordingly.

Pharmacologic therapy: Inflammation-driven motility abnormalities may respond to topical corticosteroids or biologic therapies targeting eosinophilic inflammation.^[36]^ Improvement in motility following treatment supports the hypothesis that inflammation plays a reversible role in motility dysfunction.^[37]^

Dietary intervention: Elimination diets may improve symptoms and histology, but their effect on motility remains less well defined. Some studies suggest that motility abnormalities may persist despite histologic response, indicating that structural remodeling may not be fully reversible.^[38]^

Dilation: In patients with significant luminal narrowing or decreased distensibility, endoscopic dilation remains a cornerstone of management.^[39]^ However, patients with apparent motility disorders without strictures may actually have reduced compliance rather than true neuromuscular disease, and may not benefit from dilation alone, underscoring the need for individualized assessment using HRM or FLIP.^[40]^

Surveillance and follow-up: Motility assessment may also aid in long-term monitoring, particularly in patients with refractory symptoms. Serial HRM or FLIP measurements can help evaluate treatment response and guide further interventions^[41]^ as show in table 3.

**Table 3:** Clinical scenarios where motality assessment is indicated in EOE.

Clinical scenario	Recommend modality	Rational
Persistence dysphagia despite histologic remission	HRM + FLIP	Identification underlying motility dysfunction or reduced compliance
Suspected structure or remodeling with out endoscopic findings	FLIP	Detects decreased distensibility not visible on standard imagine
Symptoms overlapping with primary motility disorders	HRM + esophageal biopsy	Differentiation EoE from achalasia or EGJOO
Pre-and post treatment evaluation	HRM and/or FLIP	Assess improvement or persistent of functional abnormality
Unexplained food impaction	FLIP + HRM	Identify subtle structural or functional abnormalities affecting bolus transit

Follow-up assessment of esophageal motility using high-resolution manometry (HRM) should be appropriately timed in relation to therapeutic interventions to accurately evaluate the reversibility of functional abnormalities. Current evidence suggests that HRM is best performed after completion of induction therapy, typically around 6 to 12 weeks following initiation of topical corticosteroids or dietary elimination, when histologic remission is expected. This timing allows differentiation between inflammation-driven, potentially reversible motility disturbances and persistent abnormalities related to structural remodeling or fibrosis. In selected cases, particularly those with ongoing symptoms, repeat HRM may be warranted during long-term follow-up to assess disease progression or response to therapy.^[28]^

Importantly, esophageal motility findings should be interpreted in conjunction with endoscopic and histopathological features to provide a comprehensive assessment of disease activity. Endoscopic findings such as rings, furrows, exudates, and strictures reflect varying degrees of inflammation and remodeling, while biopsy specimens provide objective evidence of eosinophilic infiltration and subepithelial fibrosis. Reduced esophageal distensibility and abnormal motility patterns often correlate with fibrotic changes rather than active inflammation alone. Conversely, patients with active eosinophilic inflammation may demonstrate reversible motility abnormalities following treatment. Therefore, integrating HRM and FLIP findings with endoscopic appearance and histologic activity is essential for distinguishing inflammatory vs fibrostenotic phenotypes and for guiding individualized management strategies.

**Figure 1.**
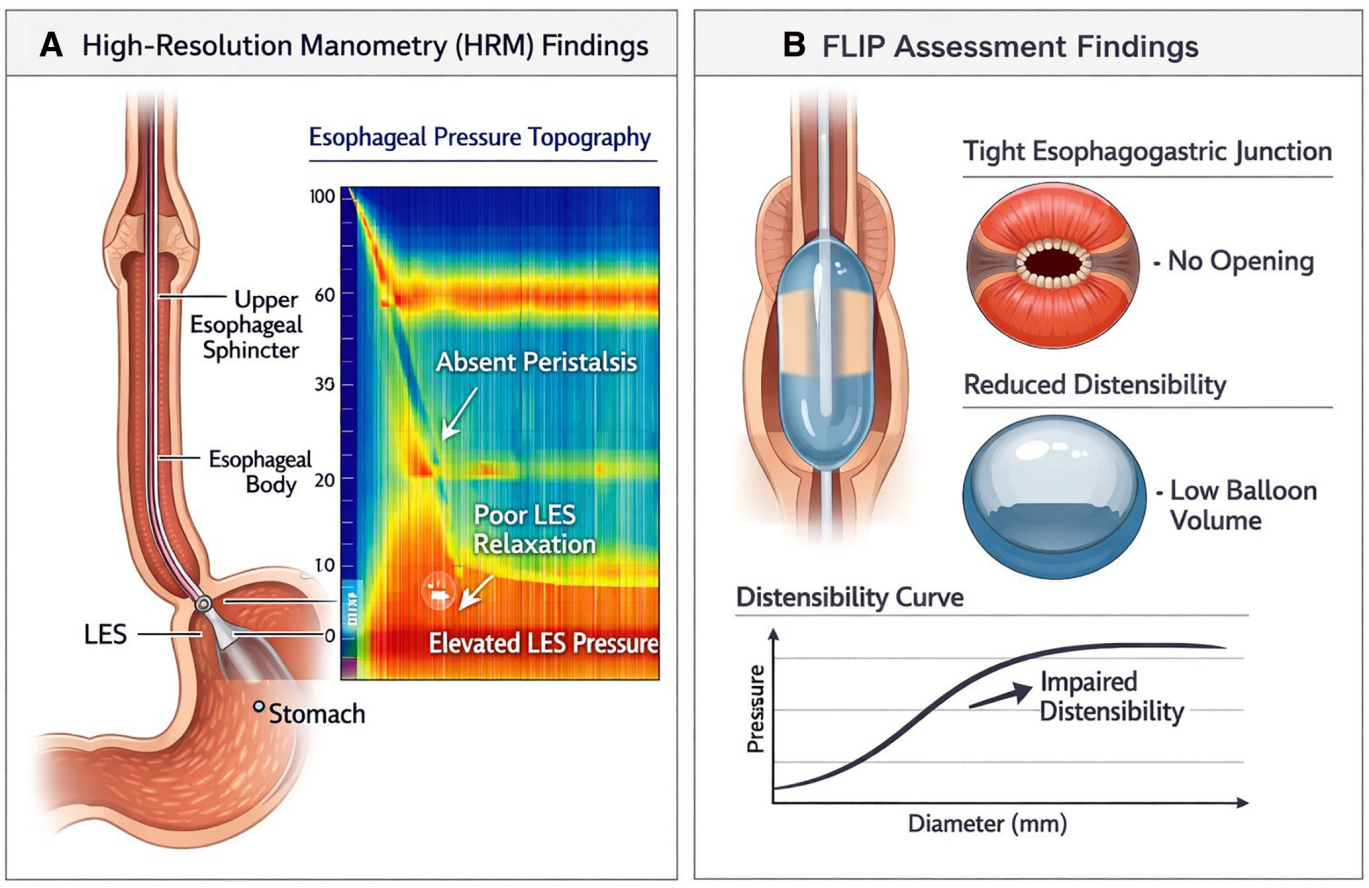
Comparative assessment of esophageal function using HRM and FLIP. (A) Illustration of the characteristic findings of high-resolution manometry (HRM) in achalasia. The anatomical landmarks include the upper esophageal sphincter (UES), esophageal body, lower esophageal sphincter (LES), and stomach. The esophageal pressure topography demonstrates absent peristalsis within the esophageal body, impaired or poor relaxation of the LES during swallowing, and elevated resting LES pressure, all of which contribute to impaired esophageal emptying. (B) Illustration of FLIP assessment of the esophagogastric junction (EGJ). The FLIP balloon catheter is positioned across the EGJ to evaluate luminal geometry and distensibility. Representative findings include a tight EGJ with minimal or no opening during balloon distension, reduced distensibility indicated by limited balloon expansion at low balloon volumes, and an abnormal pressure–diameter (distensibility) curve demonstrating impaired EGJ compliance despite increasing intraballoon pressure. Together, HRM and FLIP provide complementary assessments of esophageal motility and EGJ function in patients with achalasia. FLIP = functional lumen imaging probe, HRM = high-resolution manometry, LES = lower esophageal sphincter.

Figure 1 illustrates the complementary roles of high-resolution manometry (HRM) and FLIP in the functional assessment of eosinophilic esophagitis. While HRM provides detailed esophageal pressure topography, demonstrating abnormalities such as impaired peristalsis, elevated lower esophageal sphincter pressure, and incomplete relaxation, FLIP offers critical insights into esophageal distensibility and luminal geometry. Notably, reduced distensibility and a tight esophagogastric junction on FLIP may explain HRM patterns that resemble motility disorders, supporting the concept that some abnormalities are secondary to mechanical stiffness from subepithelial fibrosis rather than primary neuromuscular dysfunction. Therefore, the combined use of HRM and FLIP enhances diagnostic accuracy by distinguishing true motility disorders from pseudoachalasia-like physiology and provides a more comprehensive understanding of symptom generation in patients with EoE.

## 3. Future directions and research opportunities

Despite advancements, many questions remain unanswered regarding esophageal motility in EoE.^[42]^ The development of standardized protocols for HRM interpretation in EoE is needed to improve diagnostic accuracy and inter-observer agreement.^[43]^ Prospective studies are required to determine whether motility patterns can predict treatment response or long-term outcomes.^[44]^

Emerging biomarkers and imaging modalities may also help correlate histological activity with motility dysfunction.^[45]^ The role of artificial intelligence (AI) and machine learning in analyzing manometric data could offer new avenues for phenotyping and risk stratification in EoE patients.^[46]^

Furthermore, pediatric populations with EoE remain underrepresented in motility studies. Since early-onset EoE may lead to long-standing changes in esophageal function, early identification of motility abnormalities could play a role in preventing progression and fibrosis.^[47]^

## 4. Conclusion

Esophageal motility abnormalities are increasingly recognized as an important component of eosinophilic esophagitis, contributing to symptomatology and influencing management. High-resolution manometry, along with advanced tools such as FLIP and impedance measurements, offers valuable insights into the functional impact of EoE beyond histologic inflammation. The heterogeneity of motility patterns reflects the complex interplay between chronic inflammation, fibrosis, and neuromuscular dysfunction. While challenges remain in interpretation and standardization, motility assessment has clear clinical relevance in the diagnosis, monitoring, and individualized treatment of EoE. Future research aimed at refining diagnostic criteria and elucidating the pathophysiological basis of motility changes will further enhance our understanding and care of this evolving disorder.

## Author contributions

**Supervision:** Ali Karim, Mahmood ul Hassan Akhundzada, Hem Raj.

**Writing—original draft:** Ali Karim, Rabia Amir, Muhammad Bari Hassan, Mubeena Javed, Mahmood ul Hassan Akhundzada, Alfa Almani, Sajid Ali, Hem Raj, Abida Perveen.

**Writing—review & editing:** Ali Karim, Rabia Amir, Muhammad Bari Hassan, Mubeena Javed, Mahmood ul Hassan Akhundzada, Danish Kumar Goswami, Alfa Almani, Sajid Ali, Hem Raj, Aamer R Mohammad, Abida Perveen.

**Resources:** Rabia Amir, Danish Kumar Goswami.

**Software:** Rabia Amir, Alfa Almani, Sajid Ali, Aamer R Mohammad.

**Visualization:** Rabia Amir, Mubeena Javed, Danish Kumar Goswami.

**Project administration:** Muhammad Bari Hassan, Mubeena Javed.

**Validation:** Muhammad Bari Hassan, Sajid Ali, Abida Perveen.

**Conceptualization:** Mahmood ul Hassan Akhundzada.

**Investigation:** Mahmood ul Hassan Akhundzada.

**Data curation:** Danish Kumar Goswami, Alfa Almani.

**Methodology:** Danish Kumar Goswami.

**Formal analysis:** Hem Raj.
